# New Members Elected

**Published:** 1865-07

**Authors:** 


The gentlemen were unanimously elected by the Association.

The Nominating Committee reported a resolution that the several sections shall select the subjects germain to their organizations, and also appoint the committees thereon.

The Committee of Arrangements announced that Judge Warren, President of the Providence Railroad, had offered for the use of the Association a special train to Readville, for the purpose of visiting the military hospital there, at any hour that they might desire to use it. Also, that the Superintendent of the Boston public schools had caused volumes of the recent report to be sent to the State House to be distributed to such gentlemen as might wish them.

Dr. Toner, of Washington, offered an amendment to the Constitution, that delegates, on registering their names, shall pay the sum of $5, and permanent members, $3. The amendment, according to the Constitution, lies over until next year.

The Committee on Nominations, through their chairman, reported that they had decided upon Cincinnati, Ohio, as the next place of meeting of the Association. Dr. Cox, of Maryland, offered an amendment, substituting Baltimore, Md., for Cincinnati, O. A spirited debate followed, and at length the motion to amend was carried, and Baltimore decided upon as the next place of meeting.

A committee was appointed to draw up a series of resolutions on the death of Dr. S. D. Willard, late Secretary of the New York State Medical Society, and also to prepare a short biographical sketch of his life, both to be submitted to the Committee on Necrology.

A communication was received from Dr. Gross, Surgeon in charge of the military hospital at Readville, inviting the Association to visit Readville by special train on Thursday forenoon at 9 o'clock.

The convention adjourned at 2 o'clock. At the time of adjournment, the number of names registered on the books was 576, and delegates were still coming in. The sessions of the several sections were held in the afternoon.

*Social Entertainments in the Evening.*—Dr. J. Bigelow, Dr. D. H. Storer, Dr. Warren, Dr. Shattuck, Drs. Dix and H. R.

Storer, Dr. H. J. Bigelow, Dr. Upham, and Dr. Williams entertained the gentlemen of the Association, with their ladies, in an elegant and generous manner. Music, vocal and instrumental, charmed the ears of the guests, and every effort was made to make the evening agreeable and pleasant.

The residences of the gentlemen named above were visited during the latter part of the evening by Gilmore's Band, and serenaded in honor of their guests.

PROCEEDINGS OF THE THIRD DAY.

The Convention was called to order at 8¼, A.M., by the President.

On motion of Dr. Mayburry, of Pennsylvania, it was

*Resolved*, That the Permanent-Secretary be instructed to prefix to the List of Officers and Permanent Members, a list of the Ex-Presidents and Ex-Vice-Presidents of the Association.

Dr. Jarvis, of Dorchester, inquired concerning the functions of the Committee on Publication. A discussion on this question followed, in which the President, Drs. Jarvis, Sayre, of New York, Tripler, and Hibberd participated. By a vote of 45 to 37, a resolution was adopted giving the committee discriminating power.

On motion of Dr. Jarvis, of Dorchester, it was voted to establish a section on psychology.

Dr. Garrish, of New York, moved that a section on ophthalmic medicine be established.

Dr. Twitchell, of N. Hampshire, favored it. Dr. Williams, of Cincinnati, also favored the establishment of such a section, as there was need for thorough investigation of a science which had in the past ten years become, as it were, new, all the old landmarks having been swept away. The motion was opposed by Dr. Burns, of Pennsylvania, Dr. A. H. Stevens, of N. York, and others. It was finally rejected.

On motion of Dr. Hibberd, of Indiana, the section on anatomy and physiology was abolished, the subject of anatomy being assigned to the surgical section, and that of physiology to the section on hygiene.

A resolution was offered by Dr. Couper, of Delaware, and read by Dr. Tripler, of the United States Army, that this Society send a delegate annually to the convention of medical superintendents of insane establishments, and that efforts be made to cause a more intimate relation between the two organizations; the delegate to be selected by the President. Amendments to the Constitution to be acted upon at the next annual meeting were offered in relation to permanent members, delegates from local societies, and members by invitation.

